# Time-of-flight and black-blood MRI to study intracranial arteries in rats

**DOI:** 10.1186/s41747-023-00407-z

**Published:** 2024-01-09

**Authors:** Anne F. Cayron, Olivia Bejuy, Maria Isabel Vargas, Didier J. Colin, Tomohiro Aoki, Karl-Olof Lövblad, Philippe Bijlenga, Brenda R. Kwak, Eric Allémann, Sandrine Morel

**Affiliations:** 1 Department of Pathology and Immunology, Faculty of Medicine, University of Geneva, CMU, Rue Michel-Servet 1, CH-1211 Geneva, Switzerland; 2 Geneva Center for Inflammation Research, Faculty of Medicine, University of Geneva, Geneva, Switzerland; 3 School of Pharmaceutical Sciences, University of Geneva, Geneva, Switzerland; 4 Institute of Pharmaceutical Sciences of Western Switzerland, University of Geneva, Geneva, Switzerland; 5 CIBM Center for BioMedical Imaging, Faculty of Medicine, University of Geneva, Geneva, Switzerland; 6 Small Animal Preclinical Imaging Platform, Faculty of Medicine, University of Geneva, Geneva, Switzerland; 7 Division of Neuroradiology, Faculty of Medicine, Geneva University Hospitals and University of Geneva, Geneva, Switzerland; 8 Department of Pharmacology, Jikei University School of Medicine, Tokyo, Japan; 9 Division of Neurosurgery, Department of Clinical Neurosciences, Faculty of Medicine, Geneva University Hospitals and University of Geneva, Geneva, Switzerland

**Keywords:** Aneurysm, Intracranial arterial diseases, Magnetic resonance angiography, Magnetic resonance imaging, Rats

## Abstract

Intracranial aneurysms (IAs) are usually incidentally discovered by magnetic resonance imaging (MRI). Once discovered, the risk associated with their treatment must be balanced with the risk of an unexpected rupture. Although clinical observations suggest that the detection of contrast agent in the aneurysm wall using a double-inversion recovery black-blood (BB) sequence may point to IA wall instability, the exact meaning of this observation is not understood. Validation of reliable diagnostic markers of IA (in)stability is of utmost importance to deciding whether to treat or not an IA. To longitudinally investigate IA progression and enhance our understanding of this devastating disease, animal models are of great help. The aim of our study was to improve a three-dimensional (3D)-time-of-flight (TOF) sequence and to develop a BB sequence on a standard preclinical 3-T MRI unit to investigate intracranial arterial diseases in rats. We showed that our 3D-TOF sequence allows reliable measurements of intracranial artery diameters, inter-artery distances, and angles between arteries and that our BB sequence enables us to visualize intracranial arteries. We report the first BB-MRI sequence to visualize intracranial arteries in rats using a preclinical 3-T MRI unit. This sequence could be useful for a large community of researchers working on intracranial arterial diseases.

**Relevance statement** We developed a black-blood MRI sequence to study vessel wall enhancement in rats with possible application to understanding IAs instability and finding reliable markers for clinical decision-making.

**Key points**

• Reliable markers of aneurysm stability are needed for clinical decision.

• Detection of contrast enhancement in the aneurysm wall may be associated with instability.

• We developed a black-blood MRI sequence in rats to be used to study vessel wall enhancement of IAs.

**Graphical Abstract:**

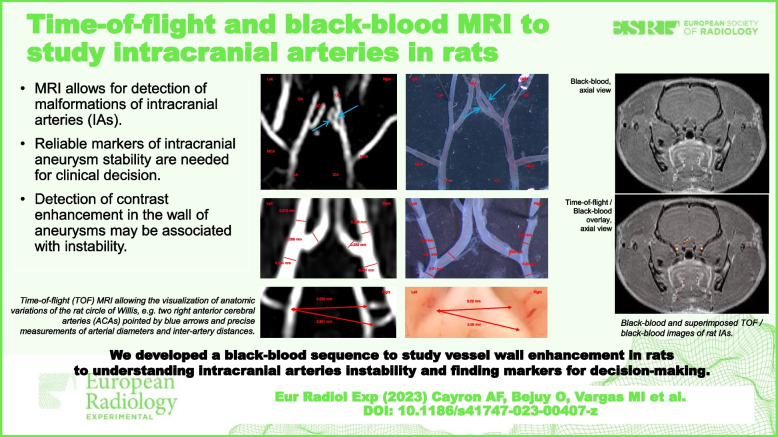

## Background

Magnetic resonance imaging (MRI) has revolutionized patient care for cerebrovascular diseases. Recent MRI developments allow for arterial wall characterization, quantification of intravascular flow, studies toward the natural history of the disease, and evaluation of treatment benefits [1]. Neurovascular examinations are commonly performed using time-of-flight (TOF) MRI sequences [1]. Intravenous injection of paramagnetic gadolinium chelates improves the contrast between the arteries and their surrounding tissues. Such contrast-enhanced magnetic resonance angiography is used to assess atherosclerotic disease, vascular dissection, and intracranial aneurysms (IAs) [2–4]. Vessel wall MRI is performed using a double inversion-recovery black-blood (BB) sequence that suppresses the signal from the blood and the cerebrospinal fluid to highlight the arterial wall [5]. Vessel wall pathology is brought out by comparison of images obtained before and after contrast agent injection, firstly to assess atherosclerotic lesions [2]. Atherosclerotic plaques showing contrast enhancement were histologically characterized by the presence of inflammatory cells, neo-vascularization, or intraplaque hemorrhage [2].

In recent years, vessel wall MRI was also used to evaluate IAs [6], which are local outpouchings of cerebral arteries affecting 3 to 5% of the general population [7]. Most of the IAs are asymptomatic and incidentally detected. The major risk is their rupture associated with a high level of morbidity and mortality [8]. The first use of vessel wall MRI in the context of IAs was performed to identify the culprit aneurysm for subarachnoid hemorrhage [9].

**Fig. 1:**
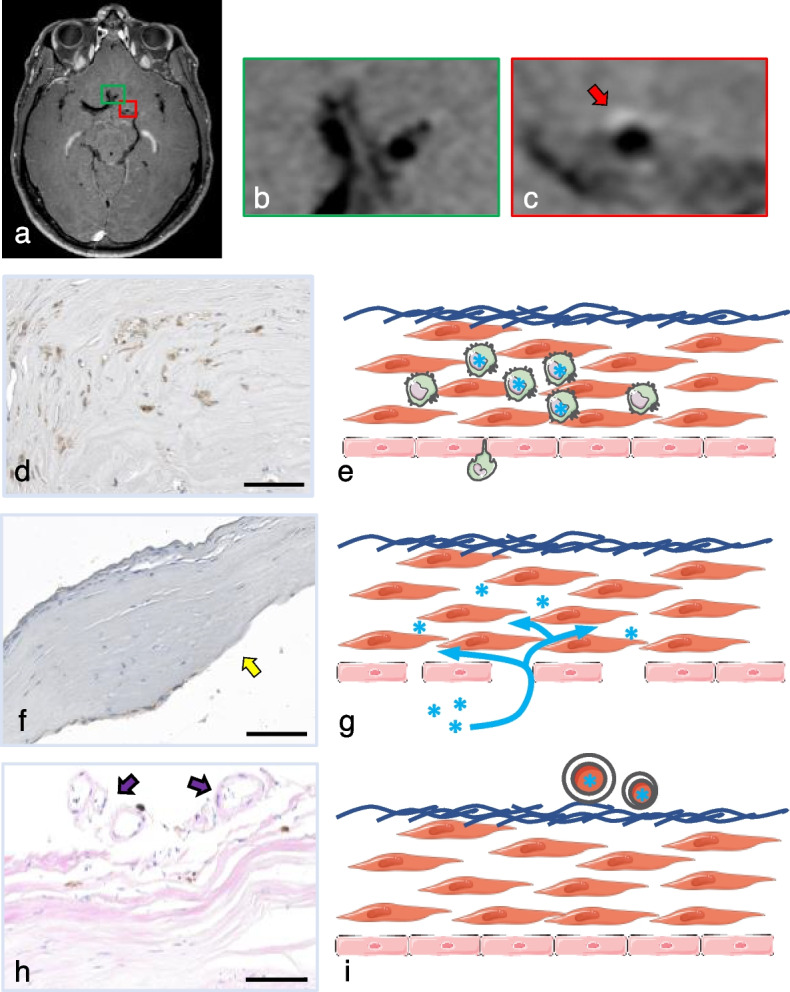
Magnetic resonance imaging vessel wall enhancement in the aneurysm wall in a 49-year-old man, former smoker, non-hypertensive, without previous history of subarachnoid hemorrhage, currently presenting for headache. **a**–**c** A red arrow shows the region with vessel wall enhancement (**c**). In comparison, normal cerebral arteries do not show vessel wall enhancement (**b**). The image was acquired at 3-T after gadolinium-chelate injection with a BB T1 sequence (field of view 210 mm, matrix 256, in-plane resolution 0.4 × 0.4 mm^2^, slice thickness 0.8 mm, 56 slices, TE/TR 20/600 ms, 3.4 averages, parallel imaging (GRAPPA) acceleration factor 2, acquisition time 6 min 23 s). Observation of AWE can be due to (1) the presence of phagocytes (**d**, representative example of CD68+ cells stained in brown) that can take up contrast agent (**e**, schematic illustration, phagocytes containing gadolinium chelate (blue asterisk); (2) compromised endothelial barrier integrity (**f**, CD31+ cells stained in brown, absence of endothelial cells indicated by the yellow arrow) leading to the passage of contrast agent from the arterial lumen into the aneurysm wall (**g**, schematic illustration); and/or (3) detection of contrast agent circulating in adventitial *vasa vasorum* (**h**, some *vasa vasorum* are indicated by purple arrows in the representative example; **i**, schematic illustration). The three representative staining examples (**d**, **f**, **h**) are from IA domes of two different patients and do not refer to the subject imaged in panels **a**–**c**. All patients consented to the @neurIST study and to the use of their images in the field of cerebrovascular research. Scale bars represent 100 μm

More recently, studies performed in humans suggest that observation of aneurysm wall enhancement (AWE), which corresponds to the presence of a certain amount of gadolinium chelate in the aneurysm wall (Fig. 1a–c), is a sign of IA wall instability [4, 10, 11]. Three hypotheses are currently proposed to explain AWE in human IA walls, *i.e.*, the excessive infiltration of phagocytes that would absorb the contrast agent (Fig. 1d, e), a compromised endothelial barrier integrity leading to contrast leakage from the lumen into the arterial wall (Fig. 1f, g), and/or detection of the contrast agent circulating in adventitial *vasa vasorum* (Fig. 1h, i) [12–14]. In clinical practice, the definition of AWE is based on four grades: (0) no or questionable focal AWE; (1) focal thick (> 1 mm) AWE; (2) thin (≤ 1 mm) circumferential AWE; and (3) thick (> 1 mm) circumferential AWE [15].

Although the BB imaging technique has furthered the visualization of the wall of intracranial vasculature facilitating clinical diagnosis, the meaning of AWE is not completely unraveled. To safely use AWE as a reliable diagnostic marker for IA instability, there is a need to deeply understand its meaning. To link the different stages of the aneurysmal disease to the different grades of AWE, it is necessary to monitor the disease during its progression from IA initiation, *via* growth to rupture. For ethical reasons, such a complete follow-up approach cannot be performed in humans and requires the use of preclinical animal models, more particularly rats, to follow the disease progression longitudinally. Until now, no BB sequences have been developed to study IA development in rats. As a first step in sequence development, our aim was to improve a three-dimensional (3D)-TOF sequence and to develop a BB sequence on a preclinical 3-T MRI to image accurately the anatomy of intracranial vessels in rats.

We herein show that our 3D TOF sequence allows for reliable measurements of intracranial artery diameters, inter-artery distances, and angles between arteries, and we report the first BB sequence to be used in rats to study intracranial arteries.

## Methods

### Animals

To perform longitudinal studies on IA initiation and growth, the preclinical IA rat model refined by the Aoki group is the most frequently used [16, 17]. As our future goal is to use this model, we have as a first step improved the intracranial 3D-TOF sequence and developed a BB sequence in healthy rats. Eleven-week-old male Sprague-Dawley rats were used for this study. The experiments were approved by the Swiss cantonal veterinary authorities (license GE21519A) and performed according to the Guide for the Care and Use of Laboratory Animals and to the Swiss national animal protection laws.

Rats were killed by an intraperitoneal injection of ketarom (ketasol 100 mg/kg and xylazine 10 mg/kg) and transcardially perfused with 4% paraformaldehyde. The brains were post-fixed for 24 h in 4% paraformaldehyde. Cerebral arteries constituting the circle of Willis were dissected from the base of the brain and images were taken using a Stemi 508 stereo microscope (Zeiss, Oberkochen, Germany).

### MRI protocol

**Fig. 2:**
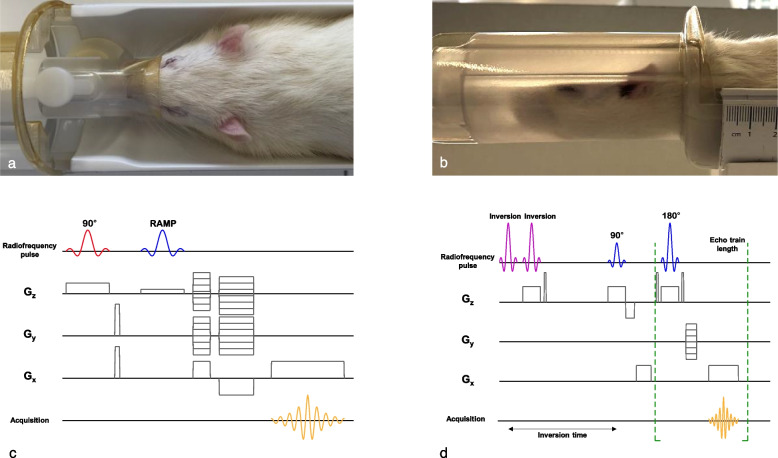
MRI bed and TOF and BB sequences for rat brain vasculature imaging. **a**, **b** Rat positioning in prone position in the MRI bed; intracranial arteries were imaged using a 3-T nanoScan® MRI unit equipped with a rat head coil. **c** Gradient-echo TOF with three-dimensional encoding mode, mainly used for unenhanced TOF angiography. Before the excitation, a slab selective saturation pulse was applied (90° RF) with selection gradient in *z* direction (G_z_) and spoiler gradients. The saturation band was placed above the imaging volume. After the saturation, a gradient echo encoding and readout came with an excitation RF pulse, phase encodings, and readout. A “RAMP” excitation pulse was used where the flip angle increases linearly along the direction of flow. Additionally, flow compensation was applied as well to eliminate artifacts caused by blood flow. The compensation gradients were placed after the RF pulse and reset the moment of all applied gradients. **d** Two-dimensional BB fast spin-echo with double inversion: the first inversion pulse was nonselective, impacting the whole volume, the second one, slice selective; the echo train of spin echo filled the *k* space; spin-echo RF pulses were slice selective. *BB* Black-blood, *G*_*x*_ Gradient in *x* direction, *G*_*y*_ Gradient in *y* direction, *MRI* Magnetic resonance imaging, *RAMP* Ramped, *RF* Radiofrequency, *TOF* Time-of-flight

Rats were anesthetized with 4% isoflurane. A catheter prefilled with NaCl/heparin was introduced into the tail vein to allow for contrast agent injection. Rats were placed in prone position in the MRI bed (MultiCell rat Brain Imaging Chamber, Mediso Medical Imaging Systems, Budapest, Hungary, Fig. 2a, b), and an ocular gel was applied to avoid dryness. A respiratory probe was placed under the rib cage to monitor breathing (Pneumatic sensor, Mediso Medical Imaging Systems, Budapest, Hungary). Animals were imaged with a 3-T nanoScan® MRI (Mediso Medical Imaging Systems, Budapest, Hungary), equipped with a 100% cryogen-free superconducting magnet with 170-mm bore size. The experiments were conducted with a dedicated brain quadrature radiofrequency coil having an inner diameter of 48 mm (Rat head coil 3.0T, Mediso Medical Imaging Systems, Budapest, Hungary). During imaging, anesthesia was set to 2−3% isoflurane to keep breathing frequency around 60 breaths/min, and body temperature was maintained at 37 °C by a flow of warm air.

Three sequences were performed:

1. 3D-TOF gradient-echo sequence with flow compensation (8 excitations; repetition time 15.1 ms; echo time 6.2 ms; flip angle 40°; in-plane resolution 0.200 × 0.250 mm; field of view in *z* axis 38 slices × 0.2 mm; slab thickness 7.6 mm; no slice gap; imaging time 12 min (Fig. 2c))
2. BB double-inversion-recovery spin-echo sequence (12 excitations; repetition time 1,200 ms; inversion time 400 ms; echo time 8.2 ms; echo train length 4; echo spacing 8.22 ms; matrix 360 × 340; in-plane resolution 0.100 × 0.141 mm; field of view in *z* axis 16 slices × 0.6 mm; slab thickness 9.6 mm; no slice gap; imaging time 30 min (Fig. 2d))
3. Same BB sequence after gadolinium-based contrast injection (Dotarem®, Guerbet AG Zurich, Switzerland) via the tail vein catheter (0.6 mmol/kg; manual injection)

### MRI and microscope image analysis

MRI and microscope images were processed using Horos software (Horos v3.0, Horos project, Brooklyn, New York, United States of America) and ImageView software (ImageView v4.11.18709.20210403, provided with the Stemi 508 stereo microscope, Zeiss, Oberkochen, Germany), respectively.

Intracranial arteries diameters and inter-artery distances were measured on TOF and microscope images. Bending of the olfactory artery (OA) and the anterior cerebral artery (ACA) bifurcation have been described and correlated with IA development [16]. According to this article, three defined angles were measured to characterize the bending of the bifurcation: (A) angle between the proximal parent artery and the OA; (B) angle between the proximal parent artery and the ACA; and (C) angle between the OA and the ACA (daughter-daughter (DD) angle). From the angles A and B, the parent-daughter (PD) angle was calculated using the following formula: PD = 180 − (A + B)/2.

## Results

**Fig. 3:**
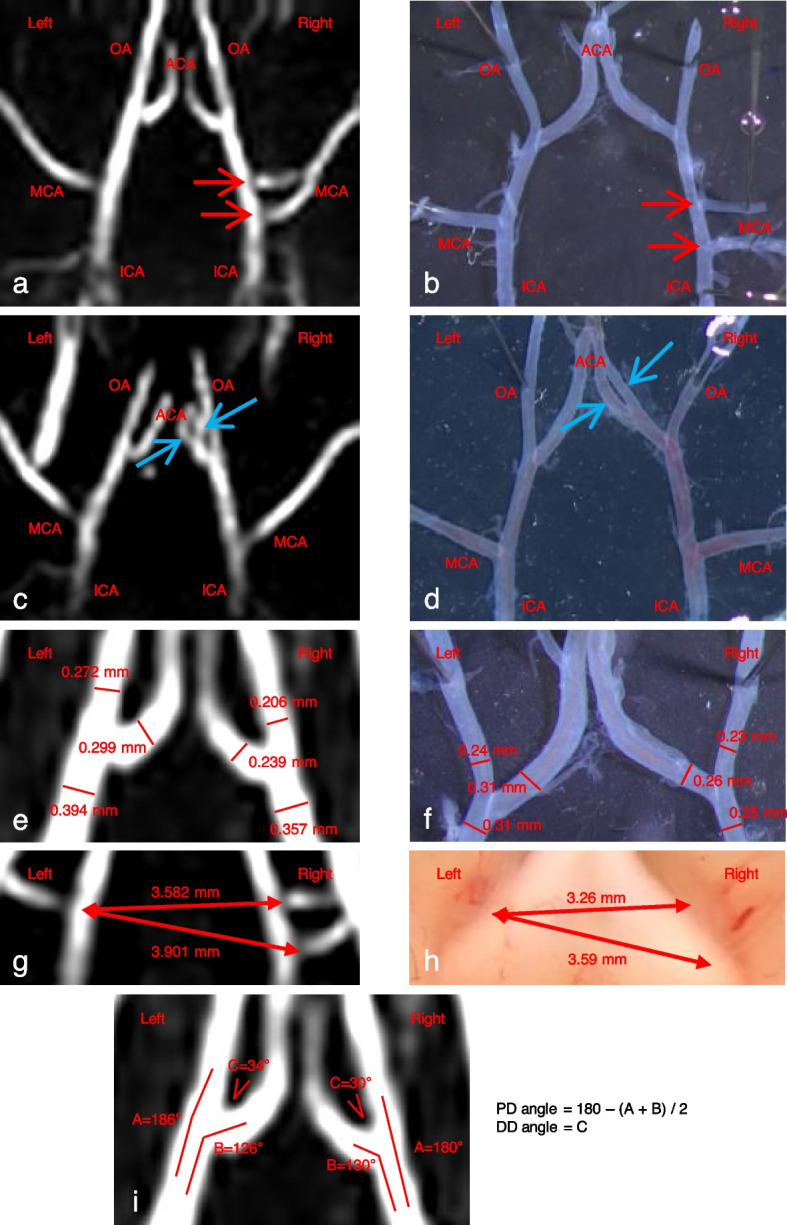
Reliability of TOF images in rats. TOF images (**a**, **c**) and microscope (**b**, **d**) images show the right and left bifurcation sites of the ACA, OA, ICA, and MCA. These two representative examples show anatomic variations of the circle of Willis: **a**, **b** rat with two right MCAs pointed by red arrows; **c**, **d** rat with two right ACAs pointed by blue arrows. Intracranial artery diameters measured on TOF image (**e**) and on pictures taken with the microscope (**f**). Distances between right and left MCA measured on TOF images (**g**) and on pictures taken with the microscope (**h**). Arteries angles at the OA, ACA, and MCA bifurcations measured on TOF image (**i**). The body weight of these two animals was 390 and 450 g. *ACA* Anterior cerebral artery, *DD* Daughter-daughter angle, *ICA* Internal carotid artery, *MCA* Middle cerebral artery, *OA* Olfactory artery, *PD* Parent-daughter angle, *TOF* Time-of-flight

3D-TOF images allowed us to clearly distinguish the right and left bifurcation sites of the ACA and OA and of the internal carotid artery (ICA) and middle cerebral artery (MCA) (Fig. 3). The two 3D-TOF examples (Fig. 3a, c) show that ramifications of the arterial tree (*i.e.,* anatomical variations) observed under microscope (Fig. 3b, d) can also be clearly seen with our TOF images.

Measurements of arterial diameters performed on TOF (Fig. 3e) and microscope (Fig. 3f) images were very similar. The average ratio of measurements made on TOF to microscope images was 1.10 ± 0.21 (mean ± standard deviation), which represents an absolute difference of 0.05 ± 0.04 mm between TOF and microscope images. Distances measured between the right and left MCA bifurcations also showed a perfect correlation between the measurements done on TOF (Fig. 3g) and microscope (Fig. 3h) images. The average ratio of TOF to microscope images for distances was 1.09 ± 0.01, which corresponds to a difference between the two modalities of less than 10%.

On our 3D-TOF images, as shown in Fig. 3i, the measures of angles defined in the “Methods” section according to Ikedo et al. [16] were as follows: left PD angle 26°; right PD angle 25°; left DD angle 34°; and right DD angle 30°.

**Fig. 4:**
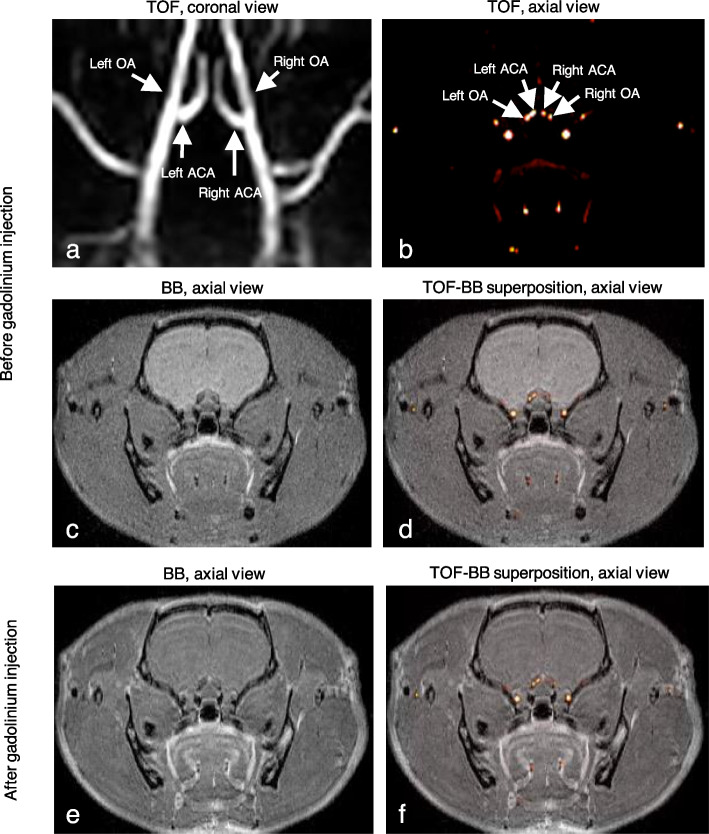
Black-blood images in rats. TOF (**a**, **b**), BB (**c**, **e**), and superposed TOF-BB (**d**, **f**) images before (**a**–**d**) and after (**e**, **f**) gadolinium-chelate injection. *BB* Black-blood, *TOF* Time-of-flight

Figure 4a and b show TOF coronal and axial views of the OA-ACA right and left bifurcations. BB axial views before and after contrast injection are shown in Fig. 4c and e, respectively. The good superposition of TOF and BB axial view before and after Gadolinium injection is shown in Fig. 4d and f, respectively.

## Discussion

In humans, contrast enhancement in the aneurysm wall observed using BB sequences is considered a sign of IA wall instability [12–14]. The pathophysiology of AWE is not entirely understood. Rat models have been established to longitudinally investigate the progression of IA disease [17]. Here, we improved a 3D-TOF sequence and developed the first BB sequence on a standard preclinical 3-T MRI unit, with the goal to longitudinally investigate the IA wall in rats. For this reason, we have focused on the anterior part of the circle of Willis where IAs develop using a frequently used IA rat model [17, 18].

As complete longitudinal follow-up of IA disease is not possible in humans, reliable *in vivo* imaging in rats is of utmost importance to increase our knowledge of the evolution of intracranial arterial diseases. Moreover, animal models allow for the precise comparison of imaging data and histological investigations at different time points. Here, we improved a TOF sequence and demonstrated that this sequence allows for reliable measurements of intracranial arteries. We carefully compared arterial diameter and inter-arterial distances between TOF and microscopic images and showed that such measured parameters are well-comparable between the two modalities.

Using a preclinical 7-T MRI, Ikedo et al. [16] recently defined angle measurement to study changes in arterial morphology during IA initiation/growth. We demonstrated that our measurements obtained using a more widely available preclinical 3-T MRI were very close to the measures obtained with their 7-T preclinical MR system (*i.e.,* PD angle = 27.9° ± 2.9°; DD angle = 36.9° ± 2.7° [16]) confirming the reliability of our images using a more widely available 3-T MRI for animals.

In recent years, AWE is increasingly used as a sign of IA wall instability [12–14]. Additional knowledge on the pathophysiology of AWE is needed to safely decide whether AWE can be used in clinical practice as a surrogate marker for IA risk of rupture, and thus, whether IAs showing enhancement need to be secured or not. Here, we developed the first BB-MR sequence suitable for rat intracranial artery imaging using a commonly used preclinical 3-T MRI. With this sequence, the observation of the appearance of AWE at the level of rat intracranial arteries would be feasible. Moreover, the duration of the sequence (30 min) permits repetition of this imaging several times on the same animal without affecting its health. This new sequence represents a remarkable opportunity to link AWE and arterial histological modifications to better determine IA risk of rupture. Furthermore, as this sequence has been developed for a preclinical 3-T MRI, a large community of researchers will be able to use it not only in the context of IAs but also for other intracranial arterial diseases.

Here, we designed a BB-MR sequence to specifically observe AWE at the level of the OA-ACA bifurcation with the aim of investigating this phenomenon in a well-established rat model [17]. Such BB-MR sequence required 30 min to image the OA-ACA bifurcation. If the site of IA development is not precisely known, as is the case in less frequently used endogenous IA rat models [19], several hours would be required to image the complete circle of Willis. A too-long MR imaging could be harmful for the rats, which is the main limitation of our study. Although we improved a TOF sequence and developed a BB sequence adapted for 3-T MRI to precisely observe cerebral arteries, until now we only tested our BB sequence on healthy rats. This new TOF sequence should be further tested to evaluate its reliability to follow IA initiation and growth in longitudinal studies in IA rat model in which AWE is expected [20–22].

IA wall instability leading to rupture is proposed to be due to modification of the arterial wall composition (*i.e.*, more inflammatory cells, less smooth muscle cells, or less collagen fibers) [23–25]. The use of targeted MRI contrast agents enabled the visualization of arterial wall inflammation and thrombus in animal IA models [26–28], which could help detect IA wall modifications. In the future, such imaging could be used by clinicians to detect IA wall instability and to plan IA treatment before its rupture. Thus, the development of a BB sequence can be a relevant advancement to better understand IA disease and to test new contrast agents to allow the discrimination between stable and unstable IAs.

## Data Availability

Data sharing is not applicable to this article as no datasets were generated or analyzed during the current study. The images generated during the current study are available from the corresponding author upon reasonable request.

## Abbreviations

3D: Three-dimensional
ACA: Anterior cerebral artery
AWE: Aneurysm wall enhancement
BB: Black-blood
DD: Daughter-daughter
IA: Intracranial aneurysm
ICA: Internal carotid artery
MCA: Middle cerebral artery
MRI: Magnetic resonance imaging
OA: Olfactory artery
PD: Parent-daughter
TOF: Time-of-flight
